# Domino hepatocyte transplantation using explanted human livers with metabolic defects attenuates D-GalN/LPS-induced acute liver failure

**DOI:** 10.1186/s12967-022-03674-3

**Published:** 2022-10-20

**Authors:** Guang-Peng Zhou, Shi-Peng Li, Yi-Zhou Jiang, Jie Sun, Yu-Le Tan, Zhi-Gui Zeng, Lin Wei, Wei Qu, Li-Ying Sun, Zhi-Jun Zhu

**Affiliations:** 1 Liver Transplantation Center, National Clinical Research Center for Digestive Diseases, Beijing Friendship Hospital, Capital Medical University, Beijing, 101100 China; 2 Department of Critical Liver Diseases, Liver Research Center, Beijing Friendship Hospital, Capital Medical University, Beijing, 101100 China; 3 Clinical Center for Pediatric Liver Transplantation, Capital Medical University, Beijing, 101100 China

**Keywords:** Domino hepatocyte transplantation, Acute liver failure, Inherited metabolic liver disease, Human primary hepatocyte

## Abstract

**Background:**

Explanted livers from patients with inherited metabolic liver diseases possess the potential to be a cell source of good-quality hepatocytes for hepatocyte transplantation (HT). This study evaluated the therapeutic effects of domino HT using hepatocytes isolated from explanted human livers for acute liver failure (ALF).

**Methods:**

Isolated hepatocytes were evaluated for viability and function and then transplanted into d-galactosamine/lipopolysaccharide-induced ALF mice via splenic injection. The survival rate was analyzed by the Kaplan–Meier method and log-rank test. Liver function was evaluated by serum biochemical parameters, and inflammatory cytokine levels were measured by ELISA. The pathological changes in the liver tissues were assessed by hematoxylin–eosin staining. Hepatocyte apoptosis was investigated by TUNEL, and hepatocyte apoptosis-related proteins were detected by western blot. The localization of human hepatocytes in the injured mouse livers was detected by immunohistochemical analyses.

**Results:**

Hepatocytes were successfully isolated from explanted livers of 10 pediatric patients with various liver-based metabolic disorders, with an average viability of 85.3% ± 13.0% and average yield of 9.2 × 10^6^ ± 3.4 × 10^6^ cells/g. Isolated hepatocytes had an excellent ability to secret albumin, produce urea, uptake indocyanine green, storage glycogen, and express alpha 1 antitrypsin, albumin, cytokeratin 18, and CYP3A4. Domino HT significantly reduced mortality, decreased serum levels of alanine aminotransferase and aspartate aminotransferase, and improved the pathological damage. Moreover, transplanted hepatocytes inhibited interleukin-6 and tumor necrosis factor-α levels. Domino HT also ameliorates hepatocyte apoptosis, as evidenced by decreased TUNEL positive cells. Positive staining for human albumin suggested the localization of human hepatocytes in ALF mice livers.

**Conclusion:**

Explanted livers from patients with inheritable metabolic disorders can serve as a viable cell source for cell-based therapies. Domino HT using hepatocytes with certain metabolic defects has the potential to be a novel therapeutic strategy for ALF.

## Background

Acute liver failure (ALF) is a rare, potentially devastating condition with jaundice, coagulopathy, hepatic encephalopathy, and multiple-organ failure. Liver transplantation (LT) has emerged as a well-established therapeutic option for ALF [1]. The widespread availability of LT and improved intensive care have reduced the overall mortality rate of patients with ALF from 80 to 30% [2]. However, the severe shortages of donor organs, high medical costs, and the need for life-long immunosuppression have become a massive barrier to patients receiving liver transplants. More importantly, it was reported that approximately 20% of patients with ALF might spontaneously recover through their native liver regeneration without the need for LT, namely spontaneous survivors [3].

Cell-based therapy seems to be a potentially promising alternative to LT, which could temporarily support and bridge patients to recovery until the regeneration of their native livers or to an available transplanted liver [4]. Previous preclinical and clinical evidence has demonstrated the promising therapeutic benefits of stem cell therapy in the treatment of end-stage liver disease [5]. Although stem cell therapy presents excellent therapeutic potential, some safety concerns remain, mainly related to the potential risk of malignant transformation and their immature phenotype [5, 6]. Against this background, mature primary hepatocytes are still considered the most desirable cell type for clinical hepatocyte transplantation (HT) [7]. However, the current sources of hepatocytes used for clinical HT are mainly donated livers that cannot be used for LT because of prolonged cold or warm ischemia, severe steatosis, etc. Correspondingly, the quality of isolated hepatocytes is often poor, thereby diminishing the therapeutic effect of clinical HT [8, 9]. Thus, there is an urgent requirement to seek alternative cell sources to resolve the shortage of high-quality hepatocytes.

In 2012, Stephenne et al. reported that a pediatric patient suffering from severe tetrahydrobiopterin nonresponsive phenylketonuria underwent two HTs using hepatocytes isolated from an explanted liver of a liver transplant recipient with glycogen storage type 1b. Blood phenylalanine levels returned to normal within three months after transplantation [10]. Gramignoli et al. found that compared with organ donors, hepatocytes isolated from the discarded liver of patients with liver-based metabolic disorders performed as well or better in terms of viability and function [11]. Taken together, explanted livers from liver transplant recipients with noncirrhotic inherited metabolic disorders of the liver may be a viable cell source for cell therapy [12]. Here, we aimed to investigate the therapeutic effects of domino HT utilizing hepatocytes with certain metabolic defects for the treatment of D-galactosamine/lipopolysaccharide (D-GaIN/LPS)-induced ALF mice. The results initially support our hypothesis that explanted livers from patients with inheritable metabolic disorders can serve as a viable cell source for cell-based therapies. Domino HT utilizing hepatocytes with certain metabolic defects might represent a potential novel cell therapeutic strategy against acute liver injury.

## Materials and methods

### Isolation, culture, and characterization of human primary hepatocytes

**Fig. 1:**
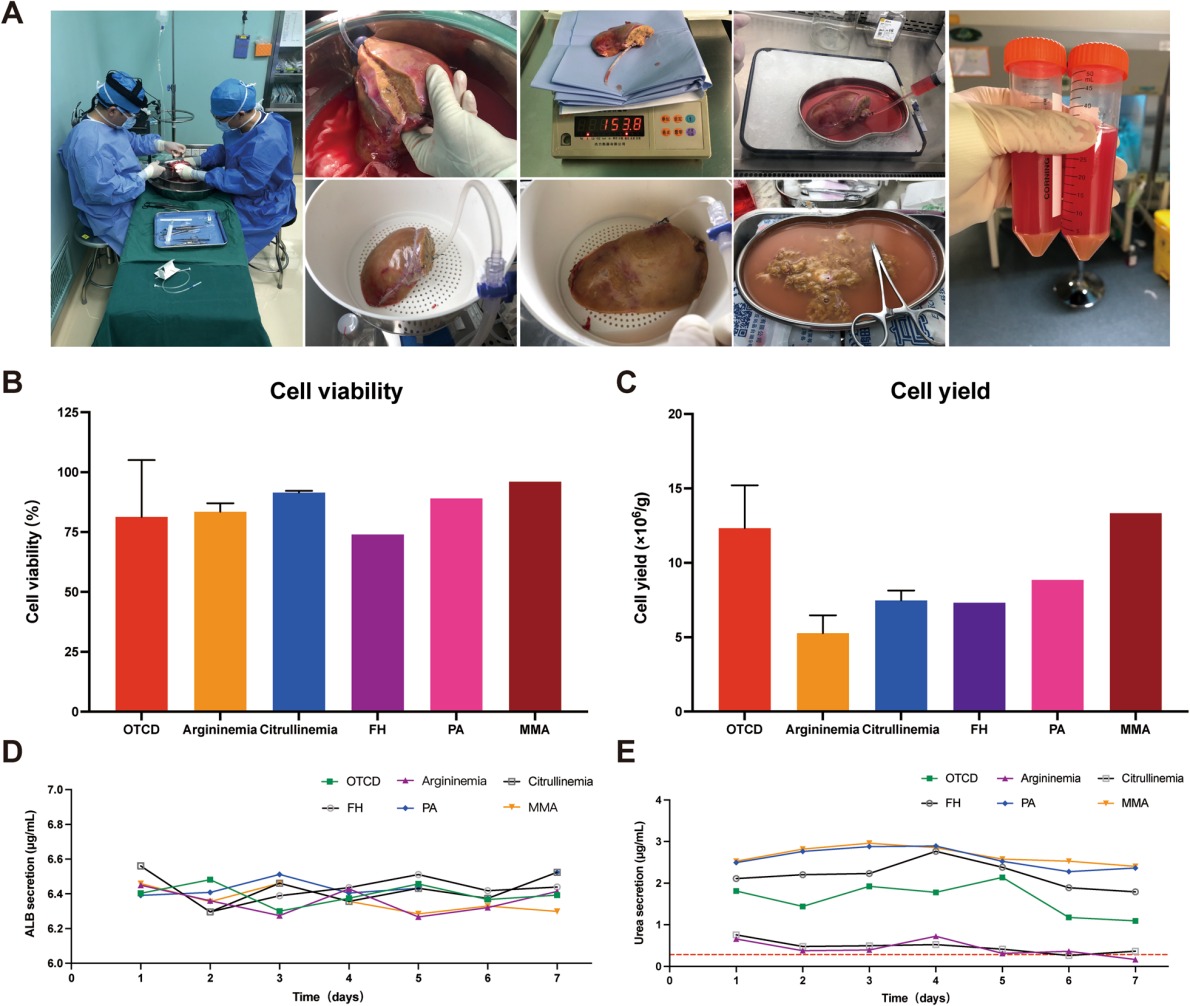
**A** Procedures for isolating hepatocytes from explanted livers of liver transplant recipients with noncirrhotic metabolic liver diseases. **B** The cell viability was assessed by the Trypan blue exclusion test. **C** The cell yield was normalized to millions of viable cells per gram of donor liver tissue. **D** Isolated human hepatocytes were able to synthesize ALB during a week of in vitro culture. **E** Isolated human hepatocytes were able to synthesize urea during a week of in vitro culture, but hepatocytes isolated from argininemia and citrullinemia produced fewer amounts of urea when compared to human hepatocytes with other metabolic defects. *ALB* albumin, *FH* familial hypercholesterolemia, *MMA* methylmalonic acidemia, *OTCD* ornithine transcarbamylase deficiency, *PA* propionic acidemia

Explanted liver from the patient undergoing LT for noncirrhotic inherited metabolic liver disease was immediately perfused with an ice-cold storage solution for solid organ preservation (Celsior® Cold Storage Solution, Waters Medical System) containing 10,000 IU low-molecular-weight heparin. The liver was split anatomically to obtain the left lateral lobe. After weighing, the liver specimen was stored in an ice-cold storage solution and immediately transferred to the laboratory for cell isolation (Fig. 1A). Moreover, transmission electron microscopy was employed to observe the change in the ultrastructure of the liver tissue. Written informed consent was obtained from the patients or the patients' guardians before LT. The experimental protocol was approved by the Ethics Commission of Beijing Friendship Hospital, Capital Medical University (2019-P2-100-01).

As previously described, primary human hepatocytes were isolated from the liver specimen using a modified two-step collagenase perfusion technique [13]. Briefly, the liver tissue was perfused with ice-cold Dulbecco's modified Eagle medium (Gibco), flushed with wash buffer, followed by digestion buffer containing 0.05% collagenase IV (Roche). The digested liver tissue was bluntly separated using a single-use sterile scalpel. The resulting cell suspension was poured through a 100-μm sterile cell strainer followed by a 70-μm sterile cell strainer and centrifuged (50 *g*, 5 min, 4 °C) three times with subsequent washing of the cell pellet using ice-cold Dulbecco's modified Eagle’s medium (Fig. 1A). Cellular sediments were resuspended in William’s medium E (Gibco) containing 10% fetal bovine serum and 1% penicillin/streptomycin.

Cell number and viability were assessed by the Trypan blue exclusion test. Hepatocytes with > 60% cell activity can be used for subsequent experiments. Freshly isolated hepatocytes were plated on cell culture dishes (Corning) at a density of 1.0 × 10^6^/ml in a 37 °C, 5% CO_2_ incubator. The hepatocytes were cultured for 7 days with a daily change of culture medium, and culture supernatants were collected daily. Albumin (ALB) and urea concentrations in the culture supernatants were determined using an ALB enzyme-linked immunosorbent assay (ELISA) kit (Bethyl Laboratories, USA) and a urea assay kit (Bioassay System, USA), respectively. Periodic acid-Schiff (PAS) staining was carried out to determine glycogen storage function. After culturing in a 37 °C, 5% CO_2_ incubator for 24 h, the hepatocytes were ready for the following experiments. After fixation in 4% paraformaldehyde, hepatocytes were stained with a PAS staining kit (BaSO, China) and then rinsed with PBS and visualized under a light microscope. For Indocyanine green (ICG) uptake assay, the hepatocytes were incubated in a medium supplemented with 1 mg/ml ICG (Sigma–Aldrich, USA) for 1 h at 37 °C and then rinsed three times with PBS before visualization under a light microscope. The remaining hepatocytes were frozen and preserved in liquid nitrogen with the cell preservation solution.

### Animals

Six- to eight-week-old male non-obese diabetic severe combined immune-deficient (NOD/SCID) mice (weighting 20–25 g) were purchased from Beijing Vital River Laboratory (Beijing, China). All animals were housed in a specific pathogen-free, temperature-controlled environment with a 12 h light/dark cycle and fed food and water ad libitum. All experimental protocols were approved by the Institutional Animal Care and Ethics Committee of Beijing Friendship Hospital, Capital Medical University (IACUC ID: 19-2003).

Mice were randomized into the following four groups: control group, D-GalN/LPS group, D-GalN/LPS + propionic acidemia (PA)-Heps group, and D-GalN/LPS + methylmalonic acidemia (MMA)-Heps group. To establish a mouse model of ALF, NOD/SCID mice were simultaneously given an intraperitoneal injection of D-GalN (700 mg/kg, Sigma–Aldrich, USA) and LPS (5 μg/kg, Sigma–Aldrich, USA). 6 h after D-GalN/LPS injection, mice were anesthetized using sodium pentobarbital and hepatocytes (2.0 × 10^6^) isolated from explanted livers with PA or MMA were administered via spleen [14]. Blood and liver tissues were harvested at 6 h after the hepatocyte administration for evaluation of liver injury (n = 6 in each group; Fig. 3A). For serum parameter assessments, serum was separated by immediate centrifugation at 3500 rpm for 15 min and stored at − 80 °C for further experiments. Liver tissues were used for histopathological analysis, immunohistochemistry, and western blot.

### Determination of mortality

The therapeutic effects of domino HT on LPS/D-GalN-induced mortality in mice were monitored over a 24 h period. The number of dead mice was counted every two hours after LPS/D-GalN injection (n = 10 in each group).

### Biochemical analysis

The serum concentrations of alanine aminotransferase (ALT) and aspartate aminotransferase (AST) were measured with an automated biochemical analyzer (CHemray-800, Rayto Life and Analytical Sciences, China).

### Inflammatory cytokines assay

The serum levels of interleukin (IL)-6 and tumor necrosis factor (TNF)-α were detected by ELISA (Mouse IL-6 Uncoated ELISA, Mouse TNF alpha Uncoated ELISA; Invitrogen, USA) according to the manufacturer’s instructions.

### Histopathological analysis and immunohistochemistry

Fresh liver tissues were fixed in 4% paraformaldehyde and embedded in paraffin wax. The 5-μm thick paraffin sections were stained with hematoxylin and eosin (H&E) for histopathological examination. For immunohistochemical staining, after deparaffinized and rehydrated, liver sections were processed for antigen retrieval in a microwave oven with citrate buffer (0.02 mol/L, pH 5.8) and then blocked with a peroxidase-blocking reagent for 15 min. Primary antibodies against human albumin (hALB; 1:100; Abcam, UK) were added and incubated overnight at 4 °C. After washing, the sections were incubated with horseradish peroxidase (HRP)-conjugated secondary antibody (ZSGB-BIO, China) for 1 h at room temperature. The sections were visualized with 3,3′-diaminobenzidine (DAB; ZSGQ-BIO, China) and counterstained with hematoxylin. Photos were taken under a light microscope.

### TUNEL assay

Terminal deoxynucleotidyl transferase dUTP nick end labeling (TUNEL; Roche) assay was carried out to assay the level of hepatocyte apoptosis in liver tissue according to the manufacturer’s instructions. Briefly, after deparaffinized, rehydrated, and retrieved, liver sections were treated with 0.1% Triton X-100 for 8 min at room temperature, blocked with 3% H_2_O_2_, washed in PBS for 3 times, and then placed in a working solution (10% enzyme solution and 90% label solution) for 1 h at 37 ℃. 4’, 6-diamidino-2-phenylindole (DAPI; Solarbio, China) was added, and the apoptotic cells were observed under a light microscope.

### Western blot analysis

Total proteins were extracted from frozen hepatocytes and hepatic samples, and then the protein concentrations were measured by BCA protein assay (Solarbio, China). The samples were separated on SDS-PAGE and transferred to a polyvinylidene fluoride membrane. The membrane was blocked with 5% skimmed milk in Tris-buffered saline containing 0.2% Tween-20 at ambient temperature for 1 h, followed by incubation with following antibodies: GAPDH (1:5000; Proteintech, China), ALB (1:2000; Proteintech, China), CYP3A4 (1:2000; Abcam, UK), alpha 1 antitrypsin (AAT, 1:2000; Abcam, UK), cytokeratin 18 (CK-18, 1:2000; Abcam, UK), Bax (1:2,000; Proteintech, China), and Bcl-2 (1:2000; Proteintech, China). The membrane was treated with HRP-conjugated goat anti-rabbit secondary antibody (1:3000; Cell Signaling Technology, USA) for 1 h at room temperature. The protein bands were visualized with an ECL chemiluminescence solution (Millipore, USA) and imaged using the Imaging System (BIO-RAD, USA). Protein expression levels were determined by densitometry using Image J software.

### Statistical analyses

The results were expressed as average value ± standard deviation. The statistical significance between various groups was determined by Student’s *t*-test. A two-tailed *P* value < 0.05 indicated statistical significance. Prism 8 version 8.2.0 (GraphPad Software) was utilized for all statistical analysis.

## Results

### Characteristics of human hepatocytes isolated from metabolically deficient livers

**Fig. 2:**
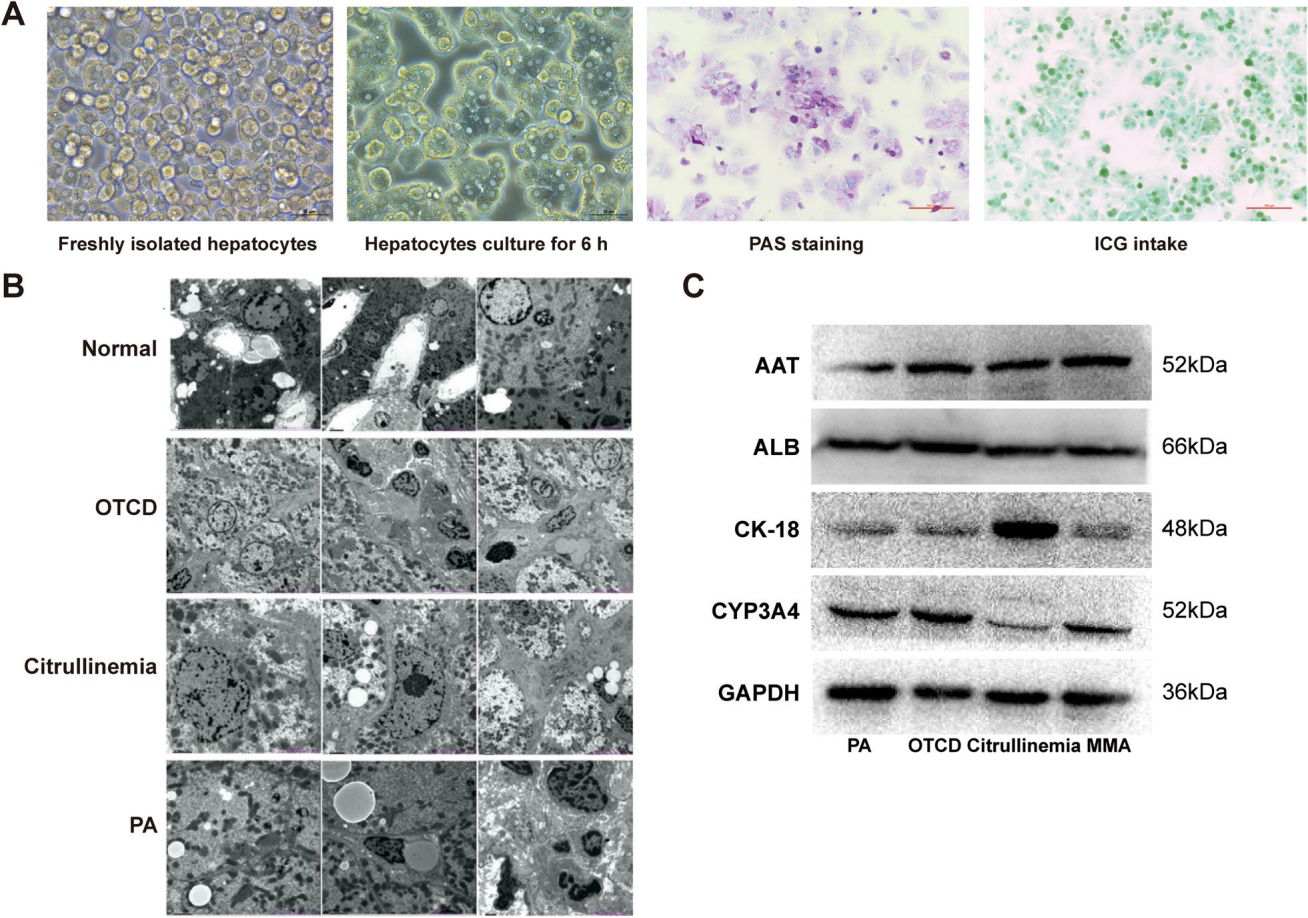
Characterization of human primary hepatocytes isolated from explanted livers of liver transplant recipients with noncirrhotic inherited metabolic liver diseases. **A** Phase-contrast microscopic images of freshly isolated and cultured hepatocytes; and PAS staining and ICG uptake assay of the cultured hepatocytes. **B** Ultrastructural features of liver tissue from normal donor and patients with noncirrhotic metabolic liver diseases under the electron microscope, magnification × 6000. **C** Protein expressions of mature primary hepatocyte markers including AAT, ALB, CK-18, and CYP3A4 of human primary hepatocytes isolated from patients with noncirrhotic metabolic liver diseases. *AAT* Alpha 1 antitrypsin, *ALB* albumin, *CK-18* cytokeratin 18, *ICG* indocyanine green, *PAS* periodic acid-Schiff

Between November 2019 and January 2021, primary human hepatocytes were successfully isolated from explanted livers of 10 pediatric patients undergoing LT for six kinds of noncirrhotic inherited metabolic liver diseases including ornithine transcarbamylase deficiency (OTCD; n = 3), argininemia (n = 2), citrullinemia (n = 2), PA (n = 1), MMA (n = 1), and familial hypercholesterolemia (FH; n = 1), with an average viability of 85.3% ± 13.0% and average yield of 9.2 × 10^6^ ± 3.4 × 10^6^ cells/g (Table 1; Fig. 1B-C). Electron microscopy images of liver specimens from patients with OTCD, citrullinemia, or PA revealed swollen hepatocytes, no hyperplasia or dilatation of the endoplasmic reticulum, and normal mitochondrial structure (Fig. 2B).

**Table 1:** Characteristics of the donated liver tissues and the isolated hepatocytes

Case	Diagnosis	Age (years)	Gender	Indication for LT	Weight (g)	CIT (min)	Viability (%)	Yield (× 10^6^/g)
ZXT	OTCD	4	Female	Recurrent hyperammonemia	154	269	54	14.50
LXY	MMA	2	Male	Recurrent metabolic decompensations	111	390	96	13.33
XCL	Argininemia	13	Male	Recurrent hyperammonemia and neurological damage	321	197	86	6.12
LHZ	OTCD	1	Male	Recurrent hyperammonemia	108	208	96	13.42
AJX	Citrullinemia	1	Female	Recurrent hyperammonemia	158	224	92	7.94
WYH	PA	2	Male	Recurrent metabolic decompensations	169	432	89	8.85
ZYX	Citrullinemia	6	Male	Recurrent hyperammonemia and neurological damage	172	268	91	7.01
ZJN	OTCD	2	Female	Recurrent hyperammonemia and liver function impairment	134	221	94	9.07
HXW	Argininemia	2	Female	Neurological damage	132	303	81	4.43
LJ	FH	5	Male	Severe atherosclerosis refractory to medical therapy	194	243	74	7.32

*CIT* cold ischemia time, *FH* familial hypercholesterolemia, *LT* liver transplantation, *MMA* methylmalonic acidemia, *OTCD* ornithine transcarbamylase deficiency, *PA* propionic acidemia

We measured ALB secretion, urea production, glycogen synthesis, ICG uptake, and protein expressions of AAT, ALB, CK-18, and CYP3A4 to determine whether hepatocytes with metabolic defects could assume the functional attributes of normal mature hepatocytes. The concentration of ALB in the culture medium was determined by ELISA assay. The results showed that the hepatocytes with metabolic defects could specifically secrete ALB (Fig. 1D). Biochemical analysis of urea in the culture medium revealed good urea production function of these metabolism-deficient hepatocytes, excepted for hepatocytes with argininemia and citrullinemia (Fig. 1E). PAS staining and ICG uptake assay was carried out to evaluate the glycogen storage and ICG uptake functions, respectively. The results revealed that these enzyme-deficient hepatocytes could be stained explicitly by PAS and uptake ICG (Fig. 2A). Western blot was carried out to assay the protein expressions of AAT, ALB, CK-18, and CYP3A4, and the results showed that these enzyme-deficient hepatocytes specifically expressed all these normal mature hepatocyte markers (Fig. 2C).

### Domino hepatocyte transplantation reduced mortality and attenuated liver injury in D-GalN/LPS-induced ALF mice

**Fig. 3:**
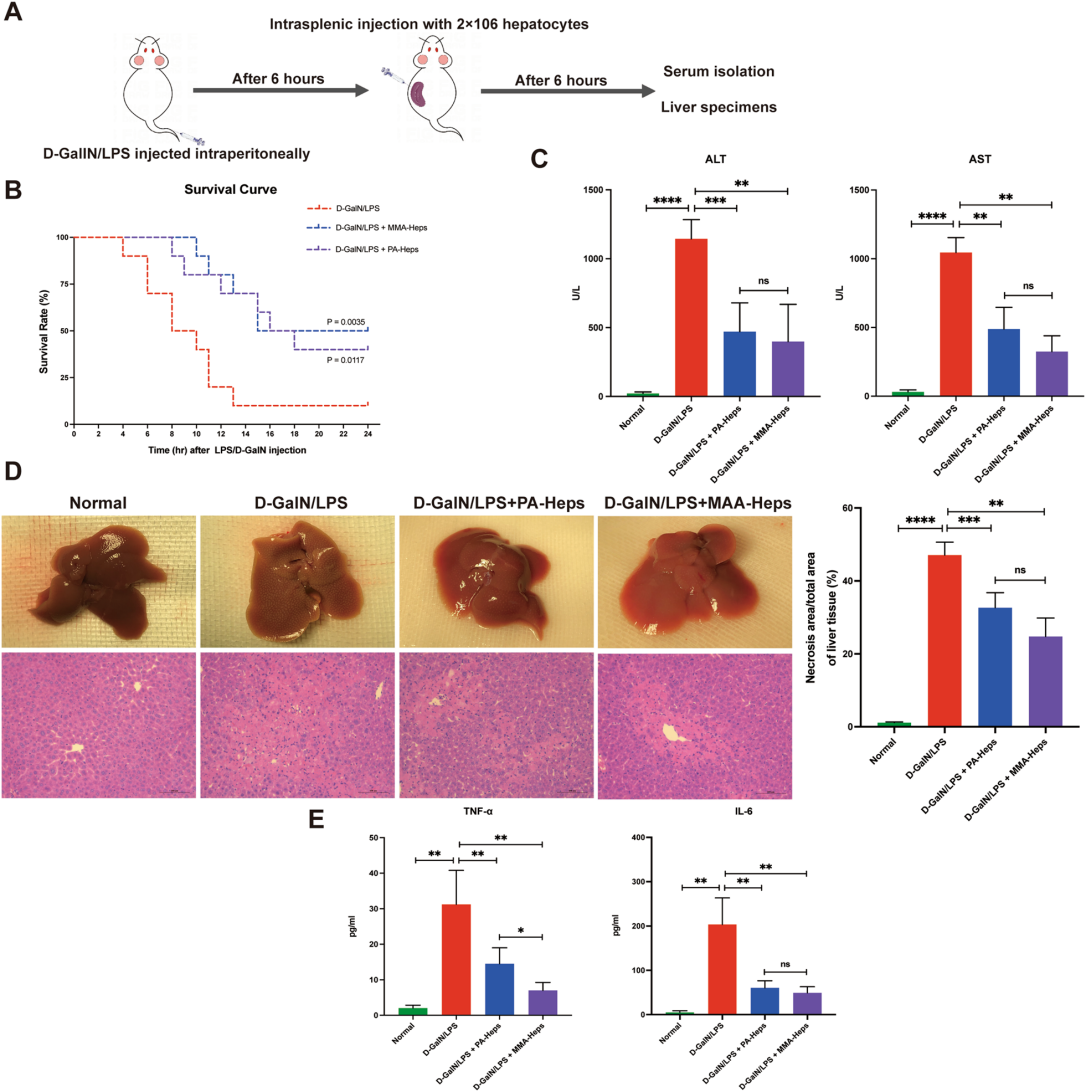
Domino hepatocyte transplantation attenuates liver injury in D-GalN/LPS-induced ALF mice. **A** Schematic representation of the study design. Mice were treated with D-GalN (700 mg/kg) and LPS (5 μg/kg) or PBS, and liver injury was assessed after 6 h. Hepatocytes (2 × 10^6^) were administered via spleen 6 h after D-GalN/LPS injection (n = 5 mice/group). **B** The survival rate of mice presented by Kaplan–Meier curve (n = 10 mice/group). **C** The serum levels of ALT and AST. **D** The gross appearance and hematoxylin–eosin staining of the collected liver tissues from the normal group, D-GalN/LPS group, D-GalN/LPS + PA-Heps group, and D-GalN/LPS + MMA-Heps group. Scale bar: 50 µm. **E** The serum levels of IL-6 and TNF-α. ns, *P* > 0.05; *, *P* < 0.05; **, *P* < 0.01.***, *P* < 0.001; ****, *P* < 0.0001. *ALF* Acute liver failure, *ALT* Alanine aminotransferase, *AST* Aspartate aminotransferase, *D-GaIN* D-galactosamine, *IL-6* Interleukin-6, *LPS* Lipopolysaccharide, *MMA* Methylmalonic acidemia, *PA* Propionic acidemia, *TNF-α* Tumor necrosis factor-α

To establish the ALF mouse model, NOD/SCID mice were intraperitoneally injected with D-GalN/LPS. Following cryopreservation and thawing, the viabilities of hepatocytes obtained from the explanted liver of a recipient with PA and MMA were 58% and 64%, respectively. The dead cells were removed using Percoll density gradient centrifugation, after which the viabilities were 92% and 93%, respectively. Hepatocytes were administered through the intrasplenic injection 6 h after GalN/LPS injection. Mice were sacrificed, and liver tissue and blood were collected 6 h after HT (Fig. 3A). To determine the therapeutic potential of domino HT in ALF mice, animal survival was observed after HT. The survival rate of hepatocyte (either from PA or MMA patients) transplanted mice was significantly greater than that of the D-GalN/LPS group (Fig. 3B). The liver function as measured by ALT and AST levels was significantly improved with domino HT compared to the D-GalN/LPS group (Fig. 3C). The gross morphology of the liver suggested that compared with the D-GalN/LPS group, hepatocyte administration reduced liver hyperemia and congestion and H&E staining also showed that the administration of hepatocyte reduces the necrosis of hepatocytes after D-GalN/LPS challenge (Fig. 3D).

ALF can cause the release of many inflammatory cytokines, such as IL-1β, IL-6, IL-10, and TNF-α. To determine the effect of domino HT on the serum levels of inflammatory cytokines in ALF mice, the serum levels of IL-6 and TNF-α were detected by ELISA. The results showed that domino HT significantly reduced the release of IL-6and TNF-α (Fig. 3E).

### Domino hepatocyte transplantation reduced hepatocyte apoptosis and inhibited expression of apoptosis-related proteins in D-GalN/LPS-induced ALF mice

**Fig. 4:**
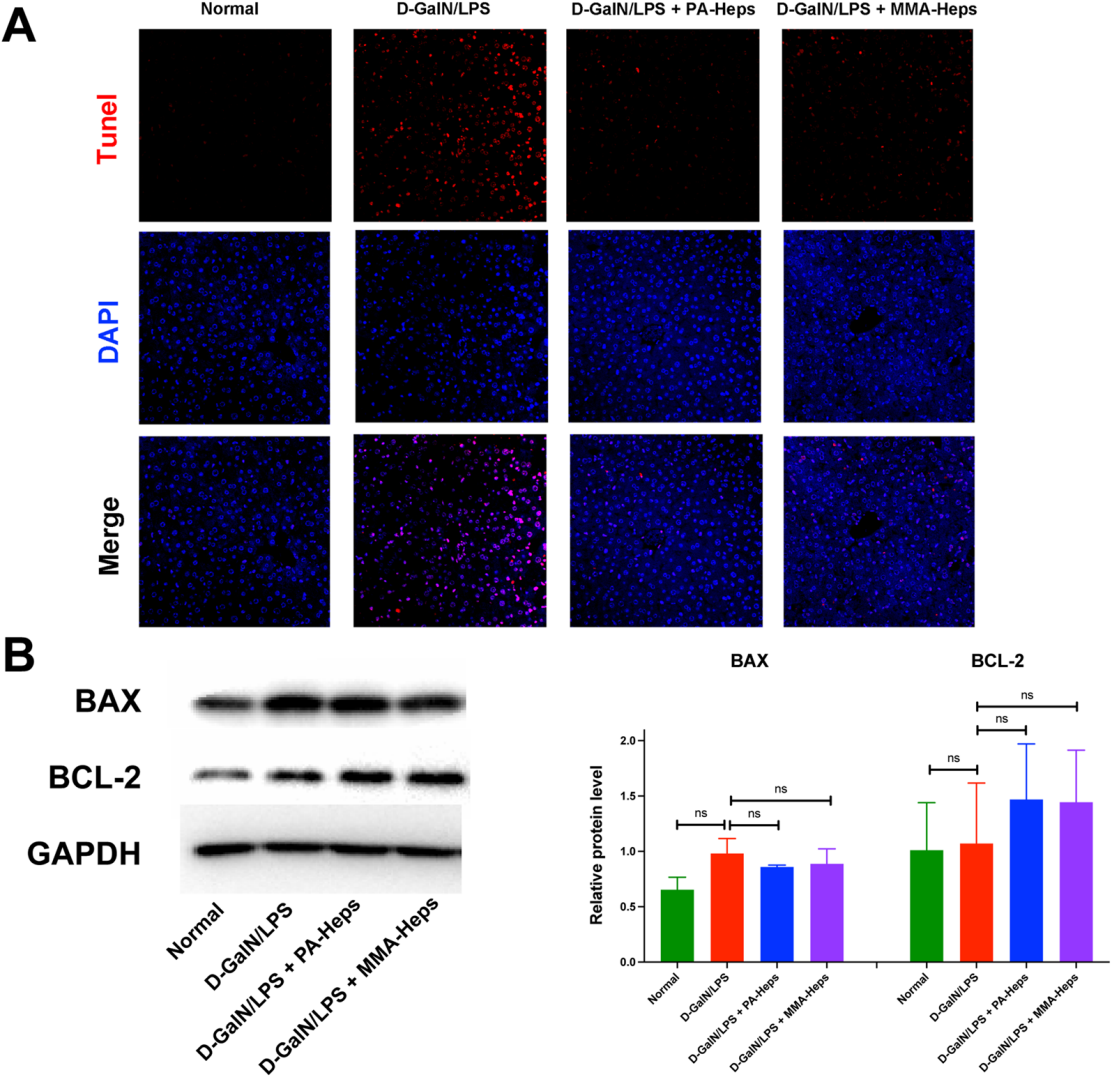
Domino hepatocyte transplantation attenuates hepatic apoptosis in D-GalN/LPS-induced ALF mice. **A** Immunofluorescence staining of liver sections of ALF mice with antibodies against TUNEL with DAPI. **B** Western blot for the expression of apoptosis-related proteins (Bax and Bcl-2) in different groups. ns, *P* > 0.05. *ALF* acute liver failure, *DAPI* *4’, 6-diamidino-2-phenylindole*, *D-GaIN* D-galactosamine, *LPS* lipopolysaccharide, *MMA* *methylmalonic acidemia*, *PA* propionic acidemia, *TUNEL* terminal deoxynucleotidyl transferase dUTP nick end labeling

To evaluate the effect of domino HT on D-GalN/LPS-induced hepatocyte apoptosis, we assessed the degree of hepatocyte apoptosis by TUNEL assay and expression of apoptosis-related signaling proteins by western blot. TUNEL assay revealed extensive hepatocyte apoptosis in the D-GalN/LPS group and a remarkable reduction of hepatocyte apoptosis after domino HT (Fig. 4A). There was no significant difference in the protein expression of pro-apoptotic protein Bax and anti-apoptotic protein Bcl-2 between groups (Fig. 4B).

**Fig. 5:**
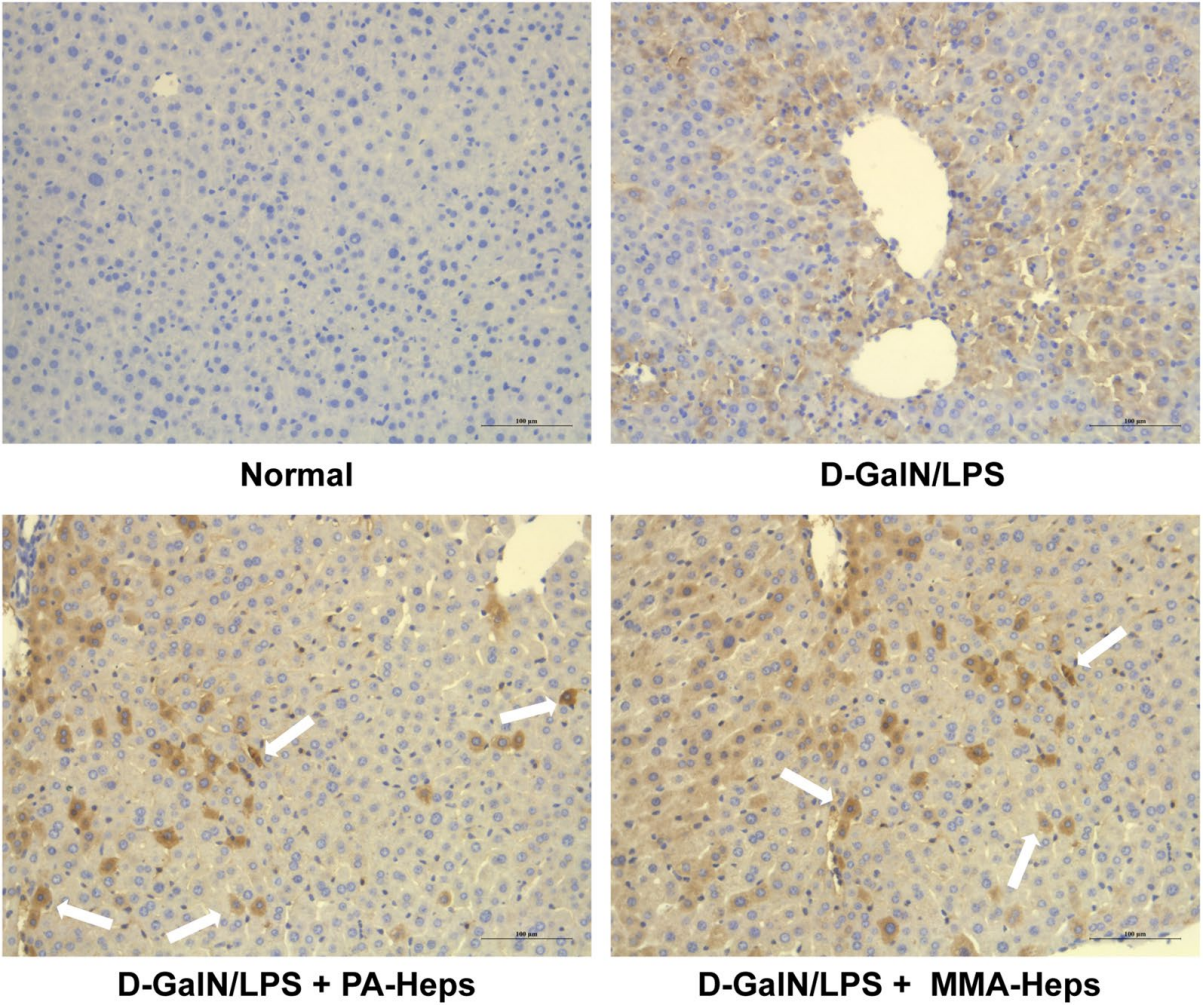
Immunohistochemical staining of hALB in mouse liver tissue. The results showed that human hepatocytes were detected in injured liver by reactivity with antibodies to hALB (white arrow), scale bar: 25 μm. *hALB* human albumin

To determine whether the transplanted hepatocytes were located in the mice liver after domino LT, immunohistochemical staining for hepatocyte-specific markers (hALB) in ALF mice liver was performed. Representative immunohistochemistry results showed the staining of hALB was positive in liver specimens of recipient mice, which confirmed the localization of human hepatocytes in *vivo* (Fig. 5).

## Discussion

Increasing clinical evidence demonstrated that HT, as an alternative or bridge to LT, is safe and feasible for the treatment of ALF [4, 15, 16]. Currently, marginal donor organs, such as livers obtained from donors after cardiac death or livers with steatosis or prolonged ischemia time, are the primary cell sources for clinical HT. However, the poor quality of these marginal donors may contribute to some unfavorable issues, including low viability and vulnerability to cryopreservation, thereby comprising the clinical therapeutic effect of HT [9]. Furthermore, the limited availability of good-quality hepatocytes has hindered the widespread application of clinical HT. Therefore, there is an urgent need to find new sources of high-quality hepatocytes.

A growing number of patients with monogenic metabolic liver diseases (such as urea cycle disorders, maple syrup urine disease, MMA, and PA) undergo LT worldwide [17–21]. The explanted livers from those liver transplant recipients with noncirrhotic liver-based metabolic disorders are morphologically, structurally, and functionally normal except for definite metabolic deficiency. Therefore, it can be reasonably speculated that hepatocytes isolated from these livers are also functionally normal except for certain metabolic defects [12]. In our study, we successfully isolated hepatocytes from discarded livers of patients transplanted for several common inborn errors of liver metabolism, including OTCD, argininemia, citrullinemia, MMA, PA, and FH. We found that these metabolically diseased hepatocytes had the properties of normal primary hepatocytes. ICG uptake assay and PAS staining showed that hepatocytes with metabolic defects possessed ICG uptake and glycogen storage functions. Western blot demonstrated expression of normal mature hepatocyte markers, including AAT, ALB, CK-18, and CYP3A4. These results were consistent with those of Gramignoli et al., who evaluated the viability and function of hepatocytes isolated from liver tissues obtained from liver transplant recipients with metabolic and other types of liver diseases and compared them with hepatocytes isolated from the donor livers rejected for LT [11]. They found that hepatocytes isolated from patients with metabolic liver diseases performed as well or better in terms of cell viability, cell yield, plating efficiency, and metabolic activity. Similarly, Bierwolf and his colleagues found that hepatocytes freshly isolated from the discarded livers of three pediatric patients suffering from different liver-based metabolic disorders (OTCD, carbamoyl phosphate synthetase deficiency, and primary oxalosis) can be used to recreate highly differentiated liver-tissue-like spheroids on alginate scaffolds [22].

Domino LT, a strategy utilizing those explanted genetically defective livers as grafts in other liver transplant candidates, has emerged as a feasible approach to increase the pool of liver grafts [23–25]. Similarly, domino HT using hepatocytes isolated from explanted livers of patients with noncirrhotic liver-based metabolic disorders may be a feasible therapeutic strategy [12, 26]. To explore whether these hepatocytes with a specific metabolic defect can be used for cell transplantation, namely domino HT, we established a D-GalN/LPS induced ALF mouse model and transplanted hepatocytes isolated from the diseased livers into ALF mice by intrasplenic injection. We found that domino HT using hepatocytes from explanted human livers significantly decreased mice mortality from ALF induced by D-GalN/LPS. The biochemical assay revealed that transplanted hepatocytes reduced ALT and AST serum levels, and liver histopathological examination showed significantly improved liver injuries after domino HT. Moreover, the serum levels of IL-6 and TNF-α significantly decreased in the treatment group. These results indicated that transplantation of hepatocytes with certain metabolic defect could attenuate liver injury and ameliorate inflammatory response in D-GalN/LPS-induced ALF mice. Collectively, our study provided preliminary evidence that domino HT could provide a time-limited protective effect against acute liver injury, and explanted livers obtained from metabolic-disordered patients could be an attractive cell source of cell-based therapy. Notably, since 10–15% of liver cell mass was required to provide hepatic function sufficient to replace the injured liver, there remain concerns about the metabolic defects secondary to transplanted hepatocytes. Thus, the enzyme defects of hepatocytes used for domino HT should be carefully considered in ALF patients. For example, given the immediate need for ammonia metabolism, we speculated that hepatocytes with urea cycle disorders-related metabolic defects are deemed unsuitable for cell transplantation for the treatment of ALF patients, which needed to be further explored [12].

It is well known that ALF and monogenic metabolic disorders are the two main indications that will benefit from HT. Mounting evidence showed that HT posed promising therapeutic value in the management of liver-based metabolic disorders [6, 7]. It was reported that 5–10% of the liver mass is required to correct the metabolic defects in recipients with monogenic metabolic disorders [6]. In the setting of domino HT, the residual native hepatocytes should be able to compensate for the metabolic defect carried by the transplanted hepatocytes. Theoretically, if the metabolic capacities and needs of the donor and recipient are carefully matched, domino HT can be a feasible treatment strategy for patients with liver-based metabolic disorders. Stéphenne et al. isolated hepatocytes from the explanted liver of a child undergoing LT for glycogen storage disease type Ib and were transplanted into a child with phenylketonuria [10]. Their results showed that although the beneficial effect was limited to a 3-month period, after two infusions, the blood phenylalanine concentrations decreased within the reference range, and the daily phenylalanine intake increased from 250 to 450 mg/day, and the half-life of phenylalanine decreased from 43 to 19 h. Despite the inspiring results, a single clinical case is underpowered to draw a definitive conclusion. More clinical cases and trials are needed to validate the therapeutic value of domino HT for patients with ALF and monogenic metabolic disorders.

Previous studies have shown that neonatal livers and remnant liver tissues obtained from living donor reduced grafts are potential sources of good-quality hepatocytes for clinical transplantation [9, 27]. However, given the scarcity of donor tissues, these cell sources are still insufficient to overcome the shortage of primary human hepatocytes. Inherited metabolic liver diseases have been the second most common indication for LT in children, accounting for 15–25% of the total after biliary atresia [28, 29], while accounts for about 5% of adult liver transplants [20]. Therefore, diseased livers removed from transplant recipients with noncirrhotic metabolic liver diseases could theoretically provide an adequate cell source for cell transplantation. Our study provided the experimental evidence of domino HT from patients with inheritable metabolic disorders to treat mice with ALF, which supports the clinical evaluation of a novel therapeutic strategy for those patients with ALF. Notably, limited engraftment and proliferation of transplanted cells remain a constraint to the generalized use of HT. Barahman et al. found that focal radiation combined with hepatic cell growth stimuli could be applied as a clinically feasible therapeutic modality to increase engraftment and repopulation of a host liver with transplanted hepatocytes [30]. Fontes et al. and Wang et al. successfully transformed lymph nodes and spleen into functioning auxiliary liver tissue, respectively, representing a potential therapeutic approach for the treatment of end-stage liver diseases [31, 32]. In addition, transplantation of alginate microencapsulated human hepatocytes has been proved to be a safe and feasible therapeutic approach in children with ALF [4]. Previous studies have shown that the molecular mechanisms of acute liver injury are related to glutathione depletion, oxidative stress, and mitochondrial dysfunction, excessive inflammatory response, and apoptosis. Therefore, novel therapeutic options improving mitochondrial functions, targeting to maintain bioenergetic status, or exerting cellular antioxidant capacity also represent potential therapeutic approaches in the treatment of ALF [33].

## Conclusions

This study suggests that explanted livers from patients transplanted for noncirrhotic inherited metabolic liver diseases can serve as a suitable alternative cell source for clinical HT, with the advantage of excellent cell viability and function. Moreover, domino HT utilizing hepatocytes with certain defined metabolic defects has the potential to be a novel therapeutic strategy for the treatment of ALF. Nevertheless, more preclinical and clinical studies are needed to verify the therapeutic value of domino HT.

## Data Availability

All data analysed during this study are included in this published article.

## Abbreviations

AAT: Alpha 1 antitrypsin
ALB: Albumin
ALF: Acute liver failure
ALT: Alanine aminotransferase
AST: Aspartate aminotransferase
CK-18: Cytokeratin 18
CIT: Cold ischemia time
DAB: 3,3′-Diaminobenzidine
DAPI: 4’, 6-Diamidino-2-phenylindole
D-GaIN: D-galactosamine
ELISA: Enzyme-linked immunosorbent assay
FH: Familial hypercholesterolemia
H&E: Hematoxylin and eosin
HRP: Horseradish peroxidase
HT: Hepatocyte transplantation
ICG: Indocyanine green
IL-6: Interleukin-6
LPS: Lipopolysaccharide
LT: Liver transplantation
MMA: Methylmalonic acidemia
NOD/SCID: Non-obese diabetic severe combined immune-deficient
OTCD: Ornithine transcarbamylase deficiency
PA: Propionic acidemia
PAS: Periodic acid-Schiff
TNF-α: Tumor necrosis factor-α
TUNEL: Terminal deoxynucleotidyl transferase dUTP nick end labeling
